# *N*-ethylmaleimide-sensitive factor interacts with the serotonin transporter and modulates its trafficking: implications for pathophysiology in autism

**DOI:** 10.1186/2040-2392-5-33

**Published:** 2014-05-10

**Authors:** Keiko Iwata, Hideo Matsuzaki, Taro Tachibana, Koji Ohno, Saori Yoshimura, Hironori Takamura, Kohei Yamada, Shinsuke Matsuzaki, Kazuhiko Nakamura, Kenji J Tsuchiya, Kaori Matsumoto, Masatsugu Tsujii, Toshirou Sugiyama, Taiichi Katayama, Norio Mori

**Affiliations:** 1 Research Center for Child Mental Development, University of Fukui, Fukui, Japan; 2 Department of Development of Functional Brain Activities, United Graduate School of Child Development, Osaka University, Kanazawa University, Hamamatsu University School of Medicine, Chiba University and University of Fukui, Fukui, Japan; 3 Research Center for Child Mental Development, Hamamatsu University School of Medicine, Hamamatsu, Japan; 4 Department of Bioengineering, Graduate School of Engineering, Osaka City University, Osaka, Japan; 5 Department of Anatomy, Hamamatsu University School of Medicine, Hamamatsu, Japan; 6 Department of Molecular Brain Science, United Graduate School of Child Development, Osaka University, Kanazawa University, Hamamatsu University School of Medicine, Chiba University and University of Fukui, Suita, Osaka, Japan; 7 Molecular Research Center for Children’s Mental Development, United Graduate School of Child Development, Osaka University, Suita, Osaka, Japan; 8 Department of Psychiatry, Hamamatsu University School of Medicine, Hamamatsu, Japan; 9 Faculty of Contemporary Sociology, Chukyo University, Toyota, Japan; 10 Department of Child and Adolescent Psychiatry, Hamamatsu University School of Medicine, Hamamatsu, Japan

**Keywords:** Serotonin transporter, NSF, Interaction, Membrane trafficking, Autism, Post-mortem brain, Lymphocyte

## Abstract

**Background:**

Changes in serotonin transporter (SERT) function have been implicated in autism. SERT function is influenced by the number of transporter molecules present at the cell surface, which is regulated by various cellular mechanisms including interactions with other proteins. Thus, we searched for novel SERT-binding proteins and investigated whether the expression of one such protein was affected in subjects with autism.

**Methods:**

Novel SERT-binding proteins were examined by a pull-down system. Alterations of SERT function and membrane expression upon knockdown of the novel SERT-binding protein were studied in HEK293-hSERT cells. Endogenous interaction of SERT with the protein was evaluated in mouse brains. Alterations in the mRNA expression of SERT (SLC6A4) and the SERT-binding protein in the post-mortem brains and the lymphocytes of autism patients were compared to nonclinical controls.

**Results:**

*N*-ethylmaleimide-sensitive factor (NSF) was identified as a novel SERT-binding protein. NSF was co-localized with SERT at the plasma membrane, and NSF knockdown resulted in decreased SERT expression at the cell membranes and decreased SERT uptake function. NSF was endogenously co-localized with SERT and interacted with SERT. While *SLC6A4* expression was not significantly changed, *NSF* expression tended to be reduced in post-mortem brains, and was significantly reduced in lymphocytes of autistic subjects, which correlated with the severity of the clinical symptoms.

**Conclusions:**

These data clearly show that NSF interacts with SERT under physiological conditions and is required for SERT membrane trafficking and uptake function. A possible role for NSF in the pathophysiology of autism through modulation of SERT trafficking, is suggested.

## Background

Autism is a pervasive developmental disorder characterized by severe and sustained impairment of social interaction and communication, and restricted or stereotyped patterns of behavior and interest. Many studies on the pathophysiological mechanisms of autism have focused on the serotonergic system. Prior studies have consistently found elevated serotonin levels in the whole blood cells and platelets of autism patients [1-5] and their relatives [6-8]. Short-term dietary depletion of tryptophan (the precursor of serotonin) has been shown to exacerbate repetitive behavior and to elevate anxiety and feelings of unhappiness in autistic adults [9]. Accordingly, many genetic studies have examined the associations between autism and genetic mutations of human serotonin transporter (SERT; solute carrier family 6 (neurotransmitter transporter), member 4 (*SLC6A4*)), especially the short allele of a polymorphism in the promoter region of the serotonin transporter gene. Although some positive relationships have been found, the results to date are inconsistent [10-15]. A single photon emission computed tomography study showed that autistic children, under light sedation, exhibit a reduction in SERT binding in the medial frontal cortex, midbrain and temporal lobe areas [16]. Importantly, our colleagues recently reported that binding of SERT and its radioligand was significantly lower throughout the brain in autistic individuals compared with controls [17]. The reduction in the anterior and posterior cingulate cortices was associated with an impairment of social cognition in autistic subjects, and a significant correlation was also found between repetitive and/or obsessive behavior and interests and a reduction in SERT binding in the thalamus [17]. These results suggested that SERT protein levels and/or its transport capacity were decreased in the brains of autistic patients. Despite this prediction, Azmitia and colleagues reported increased immunoreactivity to a SERT antibody of serotonin axons in the post-mortem cortices of autism patients [18].

SERT is an integral plasma membrane glycoprotein that regulates neurotransmission through the reuptake of 5-hydroxytryptamine (5-HT), also known as serotonin, from the synaptic cleft. SERT transport capacity is known to be regulated through mechanisms that involve subcellular redistribution of the transporter, which are regulated by various cellular mechanisms, including interactions with other proteins [19,20]. Indeed, several SERT-binding proteins have been reported. Syntaxin-1A [21-23] and secretory carrier membrane protein 2 (SCAMP2) have been reported to be associated with the N-terminal tail of SERT [24]. Macrophage myristoylated alanine-rich C kinase substrate (MacMARCKS) [25], integrin β3 [26] and nitric oxide synthase (nNOS) [27] have been reported to be associated with the C-terminal tail of SERT. SERT also forms complexes with hydrogen peroxide-inducible clone 5 protein (Hic-5) [28,29], phosphatase 2A (PP2A) [30], and α- and γ-synuclein [31,32]. By interacting with SERT, SCAMP2, MacMARCKS, nNOS, Hic-5, PP2A and α/γ-synuclein reduce the efficacy of serotonin reuptake because of a reduction in surface expression of SERT or promotion of SERT dephosphorylation [24,25,27,29-32]. Loss of integrin β3 results in decreased SERT function and surface expression in platelets [26]. Syntaxin-1A regulates the electrophysiological properties of SERT [23].

In this study, we sought to identify novel proteins interacting with the N- and C-terminal portions of SERT, and which thereby regulate SERT function. We also measured the levels of mRNAs for SERT and SERT-interacting proteins in post-mortem brains and lymphocytes from autism patients to assess their involvement in autism.

## Methods

### Animal experiments

Experiments using mice were approved by the Committee on Animal Research of Hamamatsu University School of Medicine and University of Fukui. These experiments were performed in accordance with the Guide for Animal Experimentation at the Hamamatsu University School of Medicine and the University of Fukui.

### Glutathione S-transferase pull-down assays

Full-length rat SERT complementary DNA (cDNA) was obtained from Dr Heinrich Betz (Max Planck Institute) [25,33]. PCR fragments corresponding to the N-terminal domain of the rat SERT (N-SERT; residues 1 to 85 amino acids) and the C-terminal domain of the rat SERT (C-SERT; residues 595 to 630 amino acids) were fused to glutathione S-transferase (GST) by subcloning into the pGEX-5X-1 bacterial expression vector (Amersham Bioscience, Uppsala, Sweden), to produce vectors containing GST-N-SERT and GST-C-SERT. Plasmids were transformed into *Escherichia coli* (BL21 (DE3), Stratagene, La Jolla, CA, USA) and were cultured and induced with isopropyl-β-D-thiogalactopyranoside (IPTG) at 37°C for 4 h. Mouse brain tissue was homogenized on ice using a homogenizer (Iuchi, Osaka, Japan), in 5 ml of homogenization buffer (50 mM NH_4_Cl, 40 mM Tris–HCl pH 8.0) supplemented with a 1× complete protease inhibitor cocktail (Roche Applied Science, Indianapolis, IN, USA) per brain. The same amount of extraction buffer (20 mM NaCl, 20 mM Tris–HCl pH 8.0, 1% NP-40, 1% deoxycholate) was added, and homogenates were incubated at 4°C for 30 min with rotation. Insoluble cellular debris was removed by centrifugation, and the supernatants were collected. Then, the extracts were diluted up to tenfold in homogenization buffer plus extraction buffer without detergents. Extracts were incubated with glutathione agarose bound to GST, GST-N-SERT or GST-C-SERT at 4°C for 3 h. Beads were washed five times with TBS buffer (50 mM Tris–HCl pH 7.4, 150 mM NaCl and 1 mM ethylenediaminetetraacetic acid) and boiled in SDS-PAGE sample buffer for 5 min to elute bound proteins. These samples were subjected to SDS-PAGE, which was followed by silver staining using a Silver Stain MS Kit (Wako Pure Chemical Industries, Ltd, Osaka, Japan) to visualize protein bands for mass spectrometry analysis. The samples were also used for Western blotting experiments.

### Western blot analysis

Western blotting was performed following a previously published protocol [34]. Antibodies against SERT (1:400 to 2,000; C-20, Santa Cruz Biotechnology, Inc, CA, USA), *N*-ethylmaleimide-sensitive fusion protein (NSF; 1:500; Cell Signaling Technology, Inc, Danvers, MA, USA), syntaxin-1A (1:500; Santa Cruz Biotechnology, Inc, CA, USA) or β-actin (1:1,000; Abcam Inc, Cambridge, MA, USA) were used. Immunoreactive bands were scanned and quantified using ImageJ software (ImageJ 1.44, National Institutes of Health, USA).

### In-gel digestion and mass spectrometry analysis

Protein bands were excised from SDS-polyacrylamide gels. The bands were processed in destaining solutions included in the Silver Stain MS Kit. Disulfide bonds were reduced with dithiothreitol (DTT) and the proteins were alkylated with iodoacetamide. The proteins were then treated with 50 μl (25 ng/μl) of Trypsin Gold (Promega, Madison, WI, USA) in 50 mM ammonium bicarbonate for 45 min on ice, and then overnight at 37°C. After enzymatic digestion, the peptides were eluted from the gel by treatment (twice, for 30 min each time) with 50 μl of a mixture containing 50% acetonitrile and 5% trifluoroacetic acid. The two eluates were pooled and evaporated to dryness in a vacuum centrifuge. Prior to mass spectrometric analysis, peptides were re-dissolved in 50 μl of 0.1% formic acid. Liquid chromatography-tandem mass spectrometry (LC-MS/MS) of the peptide mixtures was performed on a QSTAR XL (ESI-QqTOF; AB Sciex, Foster City, CA, USA) mass spectrometer. Product ion (MS/MS) spectra of the peptides separated by high-performance liquid chromatography (HPLC) were recorded and then submitted to the Mascot database search engine (Matrix Science [35]) for protein identification. The SwissProt database was used with ‘all entries’ for taxonomy. The tolerance was ±0.1 Da, and only one error was considered for the enzyme’s cutoff point.

### Production of a stable cell line (HEK293-hSERT cells)

The human SERT (hSERT) protein was transcribed from the human *SERT* gene. The cDNA for hSERT was isolated by RT-PCR. The PCR fragments were cloned into pcDNA3.1(+) (Invitrogen Carlsbad, CA, USA) resulting in the construct pcDNA-hSERT. To generate stably transfected cells, pcDNA-hSERT was transfected into the human embryonic kidney cell line HEK293 using Transfectamine 2000 (Invitrogen) in accordance with the manufacturer’s instructions. After 24 h, transfected cells were switched to a medium containing 1 mg/ml geneticin (G418); 1 week later, resistant colonies were isolated from culture plates using sterile clone rings. Individual cells were used to generate clonal lines. Multiple lines tested positive for immunostaining using SERT Ab (Santa Cruz Biotechnology, Inc) and a fluorescence-based uptake assay, and clonal line #7 (termed HEK293-hSERT cells) was used in all experiments reported here. The HEK293-hSERT cells were cultured in DMEM (Invitrogen) supplemented with 10% fetal bovine serum (Invitrogen), penicillin (100 U/ml), streptomycin (100 μg/ml) and G418 (0.2 mg/ml) at 37°C in 5% CO_2_.

### Primary culture of serotonergic raphe neurons

Primary culturing of serotonergic raphe neurons was performed using mouse neurons as described previously [36]. Pregnant BL6 mice (E16.5) were euthanized by cervical dislocation. Embryos were removed and placed in Hank’s balanced salt solution (HBSS) without Ca^2+^ (Life Technologies Co, Carlsbad, CA, USA). Rostral raphe neurons were dissected from the midbrain according to a method described previously [37]. Briefly, heads were removed from the embryos under a dissecting microscope (SMZ645; Nikon, Tokyo, Japan), and the midbrain/brainstem was gently dissociated. The neural tube was opened ventrally and flattened in a Petri dish containing HBSS without Ca^2+^. A strip of tissue of approximately 0.5 mm in width was dissected at the midline of the rostral rhombencephalon. Raphe tissue was resuspended in 5 ml of HBSS without Ca^2+^ and triturated ten times; the homogenate was strained through a cell strainer (BD Biosciences, Mississauga, ON, Canada) to remove debris, and an equal amount of HBSS containing Ca^2+^ was added. Cells were centrifuged (500 *g*, 5 min), and the pellet was resuspended in 5 ml of Neurobasal media (Invitrogen) containing B27 supplement (Invitrogen), penicillin (100 U/ml), streptomycin (100 μg/ml), and 0.4% l-Glutamine (Invitrogen) and plated onto eight-well slide chambers coated with poly-d-lysine (BD Biosciences). Two days after plating, 0.3 ml of medium from each well was replaced with fresh medium. Cells were cultured for 7 days *in vitro*[36].

### siRNA-mediated gene knockdown

The duplexed oligonucleotides of siRNA used in this study were based on the sequence of the human cDNA encoding *NSF. NSF* siRNAs and a non-silencing control siRNA were obtained from Integrated DNA Technologies (Coralville, IA, USA). The targeted sequences of the human *NSF* siRNAs were as follows: 5′-GGAATGCAATAAAGAGTAAATATAC-3′ (siRNA-1) and 5′-GGATAGGAATCAAGAAGTTACTAAT-3′ (siRNA-2). Transfection was performed using Lipofectamine RNAiMAX (Invitrogen) in accordance with the manufacturer’s instructions, and cells were processed 48 h after transfection.

### Immunocytochemistry and microscopy

HEK293-hSERT cells were grown on poly-d-lysine-coated glass coverslips. Raphe neurons were plated onto eight-well slide chambers coated with poly-d-lysine (BD Biosciences) and cultured for 7 days *in vitro*[36]. Cells were washed with PBS (-) and fixed with 2% paraformaldehyde in PBS (-), pH 7.4, for 15 min at room temperature (RT). Cells were washed with PBS (-) and incubated with ice cold 100% methanol for 10 min at -20°C to permeabilize them. Cells were washed with PBS (-) and incubated with blocking solution (5% skimmed milk in PBS (-)) at RT for 1 h followed by incubation with primary antibody against SERT (1:400; C-20, Santa Cruz Biotechnology, Inc), NSF (1:500; Cell Signaling Technology, Inc), cadherin (1:50; Abcam Inc, Cambridge, MA, USA) or serotonin (1:50; Gene Tex, Inc, Irvine, CA, USA) diluted in 1% skimmed milk in PBS (-) for 2 h at RT. Cells were washed in PBS (-) and incubated with the appropriate fluorophore-conjugated secondary antibody diluted in 1% skimmed milk in PBS for 60 min at RT. After washing, the cells were mounted onto microscope slides in 50% glycerol in PBS (-). Samples were imaged on a fluorescence microscope (BX53; Olympus, Tokyo, Japan) or a laser scanning confocal microscope (FluoView FV1000; Olympus).

### Fluorescence-based uptake assay

The fluorescence-based uptake assay employed a fluorescent substrate that mimics the biogenic amine neurotransmitters and is taken up by the cell through their specific transporters, resulting in increased fluorescence intensity [38]. The corresponding fluorescence-based potencies (FL pIC_50_ values) were determined in a similar manner to the [^3^H]-neurotransmitter uptake protocols [39]. HEK293-hSERT cells were plated in black, 96-well optical bottom assay plates coated with poly-d-lysine (#3882, Corning Life Sciences, Lowell, MA, USA) and transfected with siRNAs as described above. Fluorescent substrate uptake assays were performed using the Neurotransmitter Transporter Uptake Assay Kit (Molecular Devices Co, Sunnyvale, CA, USA) in accordance with the manufacturer’s instructions. Kinetic measurements of relative fluorescence units (integrated over 0.5 ms) were made using a cycle time of 5 min in a fluorescence microplate reader (SpectraMax M5; Molecular Devices Co). Data were normalized to cell number using the 3-(4,5-dimethylthiazol-2-yl)-2,5-diphenyl tetrazolium bromide (MTT) assay described below. Non-specific uptake was determined in the presence of 10 μM fluoxetine, a selective serotonin reuptake inhibitor.

### MTT assay

Cell proliferation was measured with a MTT assay. Cells were incubated with MTT solution at 37 ºC for 6 h. Following removal of the solution, dimethyl sulfoxide was added, and the amount of formazan formed was measured spectrophotometrically at 550 nm using a microplate reader (Bio-Rad, Hercules, CA, USA).

### Biotinylation

Biotinylation experiments were performed using the Cell Surface Protein Isolation Kit (Pierce, Rockford, IL, USA) in accordance with the manufacturer’s instructions. The cells were incubated with sulfo-NHS-SS-biotin solution for 30 min at 4°C, and the biotinylation of membrane proteins was stopped by adding quenching solution. The cells were washed and lysed in lysis buffer containing 1× complete protease inhibitor cocktail (Roche Applied Science). Cell lysates were incubated with NeutrAvidin Agarose beads for 1 h at RT. Beads were washed and biotinylated proteins were eluted using SDS-PAGE sample buffer. Analysis was performed on aliquots taken: (a) prior to incubation with beads (as total lysate) and (b) of the bead elute (as the biotinylated membrane fraction). Then, immunoblot analysis was carried out as described above. Analysis was performed on aliquots taken: (a) prior to incubation with beads (as total lysate) and (b) of the bead elute (as the biotinylated membrane fraction). Then, Western blot analysis was carried out as described above. For the biotinylated membrane fraction, after Western blot analysis, the membrane was stained with Coomassie Brilliant Blue (CBB) as a protein-loading control.

### Time-controlled transcardiac perfusion cross-linking and immunoprecipitation

The time-controlled transcardiac perfusion cross-linking (tcTPC) experiments were performed as described previously [40]. Mice were anesthetized and perfused with saline at 25 ml/min for 2 min to purge the blood vessels. The perfusate was switched to fixative solution (4% formaldehyde in PBS (-)) at 25 ml/min and cross-linking was carried out for 6 min. After perfusion, brains were rapidly removed from the skull, postfixed in tcTPC reagent and immediately frozen by immersion in liquid nitrogen. The perfusion and postfixing procedures were completed within 15 min. Mouse brains were homogenized on ice using a homogenizer (Iuchi, Osaka, Japan), in 5 ml of homogenization buffer (50 mM NH_4_Cl, 40 mM Tris–HCl, pH 8.0) supplemented with 1× complete protease inhibitor cocktail (Roche Applied Science) per brain. The same amount of extraction buffer (20 mM NaCl, 20 mM Tris–HCl, pH 8.0, 1% NP-40, 1% deoxycholate) was added, followed by incubation at 4°C for 30 min with rotation. Insoluble cellular debris was removed by centrifugation (3,000 rpm, 10 min), and the supernatants were then used as a brain extract. Brain extracts were pre-cleared with 30 μl of protein G-Sepharose (Thermo Fisher Scientific, Inc, Waltham, MA, USA) for 1 h at 4°C. Cleared lysates were first incubated with an anti-SERT antibody (made by two of the authors, TT and SY) at 4°C for 3 h, and then with 20 μl of protein G-Sepharose for 1 h at RT. The complex-bound resin was washed five times with IP buffer (25 mM Tris–HCl, 150 mM NaCl; pH 7.2). Immunoprecipitated complexes were boiled in 2× SDS-PAGE sample buffer for 5 min to elute bound proteins. Western blot analysis was carried out as described above.

### Post-mortem brain tissues

The ethics committee of the Hamamatsu University School of Medicine approved this study. The Autism Tissue Program (Princeton, NJ, USA) [41], the National Institute of Child Health and Human Development’s Brain and Tissue Bank for Developmental Disorders (Baltimore, MD, USA) [42] and the Harvard Brain Tissue Resource Center (Belmont, MD, USA) [43] provided frozen post-mortem brain tissues from dorsal raphe regions (*n* = 11 control and *n* = 7 autism).

### Lymphocyte samples

The participants in this study were 30 male subjects with autism spectrum disorder (ASD) and 30 healthy male controls. All participants were Japanese. They were born and lived in restricted areas of central Japan, including Aichi, Gifu and Shizuoka prefectures. Based on interviews and available information, including hospital records, diagnoses of ASD were made by an experienced child psychiatrist (TS) based on the DSM-IV-TR criteria. The Autism Diagnostic Interview-Revised (ADI-R) [44] was also conducted by two of the authors (KJT and KM), both of whom have established reliability for diagnosing autism with the Japanese version of the ADI-R. The ADI-R is a semi-structured interview conducted with a parent, usually the mother, and is used to confirm the diagnosis and also to evaluate the core symptoms of ASD. The ADI-R domain A score quantifies impairment in social interaction, the domain BV score quantifies impairment in communication, and the domain C score quantifies restricted, repetitive and stereotyped patterns of behavior and interests. The ADI-R domain D corresponds to the age of onset criterion for autistic disorder. The manual for the Wechsler Intelligence Scale for Children, Third Edition [45], was used to evaluate the intelligence quotient (IQ) of all the participants. Co-morbid psychiatric illnesses were excluded by means of the Structured Clinical Interview for DSM-IV (SCID). Participants were excluded from the study if they had any symptoms of inflammation, a diagnosis of fragile X syndrome, epileptic seizures, obsessive-compulsive disorder, affective disorders or any additional psychiatric or neurological diagnoses. None of the participants had ever received psychoactive medications before this study. Healthy control subjects were recruited locally by advertisement. All control subjects underwent a comprehensive assessment of their medical history to eliminate individuals with any neurological or other medical disorders. SCIDs were also conducted to identify any personal or family history of past or present mental illness. None of the comparison subjects initially recruited was found to fulfill any of these exclusion criteria.

This study was approved by the ethics committee of the Hamamatsu University School of Medicine. All participants as well as their guardians were given a complete description of the study, and provided written informed consent before enrollment. Whole-blood samples were collected by venipuncture from all participants. Lymphocytes were isolated from blood samples by means of the Ficoll-Paque gradient method (purity 80%) within 2 h after sampling.

### Quantitative real-time reverse-transcription-polymerase chain reaction

Total RNA was isolated from the dorsal raphe regions of post-mortem brains and lymphocytes using TRIZOL reagent (Invitrogen). The RNA samples were further purified using the RNeasy Micro Kit (QIAGEN, Hilden, Germany). First-strand cDNA was synthesized from the RNA samples using the SuperScript III First-Strand Synthesis System (Invitrogen). Quantitative real-time reverse-transcription polymerase chain reaction (qRT-PCR) analysis was performed using the TaqMan method in the ABI StepOnePlus TM Real-Time PCR System (Applied Biosystems, Foster City, CA, USA). TaqMan assay IDs of the genes are as follows: *SLC6A4*, Hs00984349_m1 and *NSF*, Hs00938040_m1. *Actin, beta* (*ACTB*; Hs99999903_m1) was used as the endogenous reference. Relative quantification of *NSF* and *SERT* expression levels in post-mortem brains was performed using the delta-delta C_T_ method [46], with the constitutively expressed gene *ACTB* as an internal control. Standard curves were constructed for *NSF*, *SERT* and *ACTB* primers to validate the application of the delta-delta C_T_ method. Relative quantification of *NSF* and *SERT* expression levels in lymphocytes was performed using the relative standard curve method, with the constitutively expressed gene *ACTB* as an internal control.

### Statistical analysis

The data were analyzed using a two-tailed unpaired *t*-test after it had been confirmed that there were no statistically significant differences in variance as assessed by the *F* test. One-way analysis of variance (ANOVA) followed by Tukey’s correction was used for multiple comparisons. One-way repeated-measures ANOVA with Tukey’s *post hoc* test was used for analysis of data from the uptake assay. The Mann–Whitney *U* test was used to evaluate differences in age, post-mortem interval (PMI) and IQs between the autism and control groups, and gene expression levels in the post-mortem brains and lymphocytes between these groups. Fisher’s exact test was used to evaluate differences in race and gender between the autism and control groups. Evaluation of the relationships between *NSF* expression level and clinical variables and symptom profiles was performed using Spearman’s rank correlation coefficient. *P* values of less than 0.05 were considered to indicate statistical significance. All statistical analyses were performed using statistical analysis software (SPSS, version 12.0 J, IBM, Armonk, NY, USA).

## Results

### Identification of *N*-ethylmaleimide-sensitive factor as a novel serotonin transporter-binding protein

**Figure 1:**
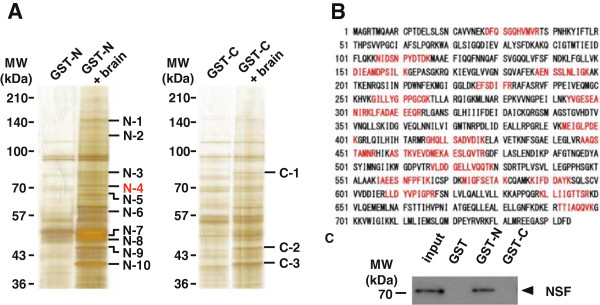
**Identification of NSF as a novel binding partner of SERT. (A)** GST-N-SERT and GST-C-SERT were incubated with and without (as negative controls) the mouse brain extract. Bound proteins were detected by SDS-PAGE and silver staining. At least ten and three specific bands were observed in the GST-N-SERT and GST-C-SERT lanes, respectively, compared with negative controls. **(B)** Analysis using Mascot identified 24 peptides (in red) that matched NSF from band N-4 (in red on (A)). **(C)** N-tail-specific binding of NSF to SERT was confirmed by Western blot analysis. C-SERT, C-terminal domain of the serotonin transporter; GST, glutathione S-transferase; GST-C, GST-C-SERT; GST-N, GST-N-SERT; N-SERT, N-terminal domain of the serotonin transporter; NSF, *N*-ethylmaleimide-sensitive factor; MW, molecular weight.

To identify novel binding proteins for SERT, we conducted pull-down experiments using GST-N-SERT or GST-C-SERT with and without (as a negative control) mouse brain lysates. After SDS-PAGE and silver staining of the gels, at least ten specific bands were observed in the lane containing proteins eluted from GST-N-SERT beads incubated with brain lysates, and at least three bands were observed in the lane containing proteins eluted from GST-C-SERT beads incubated with brain lysates (Figure 1A). The protein bands were excised from the gel and subjected to in-gel trypsin digestion. The tryptic peptide mixtures were analyzed by mass spectrometry. Excluding proteins that bound to both termini of SERT, we identified seven N-terminal-specific binding proteins, but no C-terminal-specific binding proteins (Table 1). One of the N-terminal specific bands, migrating at around 70 kDa, N-4 (Figure 1A), was identified as NSF, which regulates membrane fusion events [47,48], based on 24 independent MS spectra (Figure 1B and Table 1). We focused on the interaction between NSF and SERT in the present study for the following reasons. First, we identified NSF as having the highest reliability score (Table 1). Second, NSF interacts with neurotransmitter receptors, such as AMPA, β2 adrenergic and GABA_A_ receptors, and it regulates the membrane trafficking and synaptic stabilization of these receptors [49-57]. Finally, in the photoreceptor synapse, the NSF and Arrestin 1 interaction regulates expression of vesicular glutamate transporter 1 and excitatory amino acid transporter 5 in the photoreceptor synapse [58]. These findings suggest that NSF may interact with neurotransmitter transporters and regulates these functions in the central nervous system (CNS). To verify the interaction of NSF with SERT, we conducted Western blot analysis. GST, GST-N-SERT and GST-C-SERT were incubated with mouse brain extracts. As shown in Figure 1C, NSF bound the N-terminal region of SERT specifically. In support of previous studies, N-terminal-specific binding of syntaxin-1A was confirmed [21-23] (Additional file 1: Figure S1).

**Table 1:** Identification of GST-N-SERT and GST-C-SERT pulled-down proteins from mouse brain extracts

**Spot number**	**Gene name**	**Protein name**	**MW (Da)**	**Number**	**Sequence coverage**	**Score**	**Accession number**	**N-terminal specific**	**Cellular and molecular events**
N-1	*Synj1*	Synaptojanin 1	172,509	23	14%	785	Q8CHC4	※	Endocytosis
N-2	*Cand1*	Cullin-associated NEDD8-dissociated protein 1	136,245	14	10%	526	Q6ZQ38	※	SCF complex assembly
N-3	*Aco2*	Aconitate hydratase, mitochondrial	85,410	14	19%	534	Q99KI0		
N-4	*Nsf*	Vesicle-fusing ATPase (NSF)	82,561	24	27%	1,010	P46460	※	
N-5	*Atp6v1a*	V-type proton ATPase catalytic subunit A	68,283	13	21%	466	P50516	※	Hydrolysis
N-6	*Crmp1*	Dihydropyrimidinase-related protein 1	62,129	12	20%	441	P97427	※	Axon guidance and cell migration
N-7	*Cct2*	T-complex protein 1 subunit beta	57,441	11	19%	202	P80314	※	Molecular chaperone
N-8	*Fscn1*	Fascin	54,474	14	25%	174	Q61553	※	Actin filament binding
N-9	*Eno1*	Alpha-enolase	47,111	16	24%	703	P17182		
N-10	*Cnp*	2′,3′-cyclic-nucleotide 3′-phosphodiesterase	47,094	22	40%	341	P16330		
C-1	*Aco2*	Aconitate hydratase, mitochondrial	85,410	8	9%	287	Q99KI0		
C-2	*Eno1*	Alpha-enolase	47,111	8	20%	384	P17182		
C-3	*Cnp*	2′,3′-cyclic-nucleotide 3′-phosphodiesterase	47,094	18	32%	225	P16330		

C-SERT, C-terminal domain of the serotonin transporter; GST, glutathione S-transferase; MW, molecular weight; N-SERT, N-terminal domain of the serotonin transporter.

### Co-localization of serotonin transporter and *N*-ethylmaleimide-sensitive factor in HEK293-hSERT cells

**Figure 2:**
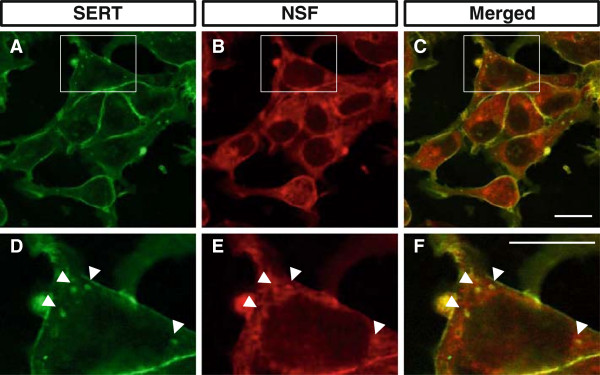
**NSF co-localizes with SERT in HEK293-hSERT cells. (A,B,C)** Double immunocytochemical staining for SERT (green) and NSF (red) in HEK293-hSERT cells. NSF co-localizes with SERT in the cell membrane (merged). **(D,E,F)** High-magnification views of the regions boxed in panels **(A)**, **(B)** and **(C)**, respectively. Arrowheads indicate double-positive intracellular particles. Scale bar: 10 μm. Results are representative of three independent experiments. NSF, *N*-ethylmaleimide-sensitive factor; SERT, serotonin transporter.

The subcellular localization of SERT and NSF was examined using immunofluorescence confocal microscopy. NSF is expressed endogenously in HEK293 cells. We established a stable human SERT-expressing cell line, HEK293-hSERT, using HEK293 cells as described in the Methods section. It was confirmed that SERT was transported to the plasma membrane in this cell line by double staining using antibodies to SERT and cadherin, a membrane marker (see Additional file 2: Figure S2). HEK293-hSERT cells were double labeled with antibodies to NSF and SERT, and it was revealed that NSF co-localized with SERT in the plasma membrane (Figure 2A,B,C) and intracellular particles (Figure 2D,E,F).

### Effect of *N*-ethylmaleimide-sensitive factor knockdown on serotonin transporter function and cellular localization

**Figure 3:**
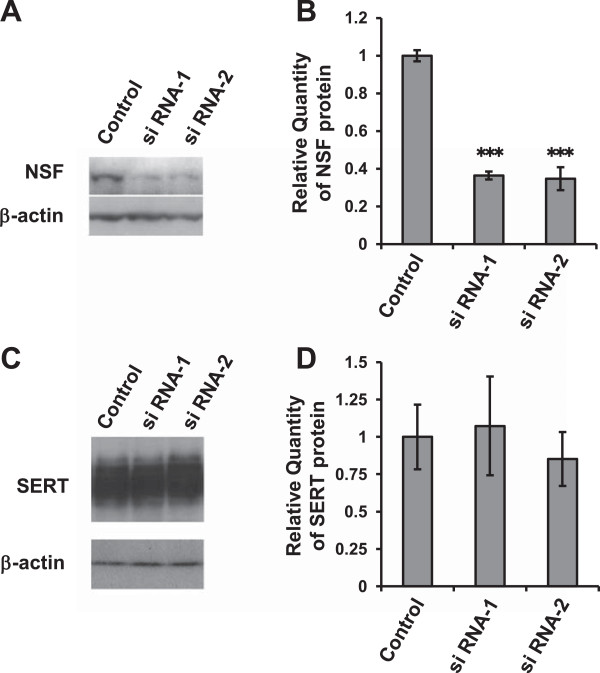
**Knockdown efficiency of NSF siRNAs and effect on expression level of SERT in HEK293-hSERT cells.** Two siRNAs targeting specific NSF sequences were transfected into HEK293-hSERT cells. **(A)** The expression levels of NSF and β-actin (as an internal control) were assayed by immunoblot analysis. **(B)** Quantitation of relative band densities for NSF was performed by scanning densitometry. Data are expressed as the means ± standard deviation, *n* = 3. ***P < 0.001 vs internal control (one-way ANOVA with Tukey’s *post hoc* test). **(C)** The expression levels of SERT and β-actin (as an internal control) were assayed by immunoblot analysis. **(D)** Quantitation of relative band densities for SERT was performed by scanning densitometry. Data are expressed as the means ± standard deviation, *n* = 5 or 6. NSF, *N*-ethylmaleimide-sensitive factor; SERT, serotonin transporter; siRNA, small interfering RNA.

**Figure 4:**
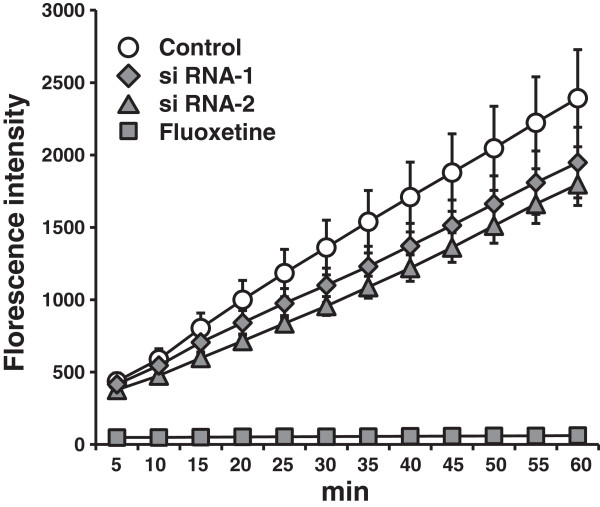
**NSF knockdown results in decreased uptake function of SERT in HEK293-hSERT cells.** Fluorescent substrate uptake activity was significantly decreased in HEK293-hSERT cells transfected with siRNAs targeting specific NSF sequences, siRNA-1 (◆) and siRNA-2 (▲), compared with negative control (○) (control vs siRNA-1 *P* < 0.01, and control vs siRNA-2 *P* < 0.001, one-way repeated measures ANOVA with Tukey’s *post hoc* test). Nonspecific uptake was determined in the presence of 10 μM fluoxetine (■). Data are expressed as a percentage of the control level. Each point corresponds to the mean ± standard deviation, *n* = 8. siRNA, small interfering RNA.

We used RNA interference to knock down endogenous NSF expression. We confirmed that the efficacy of siRNA transfection into HEK293-hSERT cells was >90% (see Additional file 3: Figure S3). As shown in Figure 3A,B, it was confirmed that both of the siRNAs (siRNA-1 and -2) targeting NSF suppressed endogenous NSF protein levels by approximately 60% (*P* < 0.001, one-way ANOVA with Tukey’s *post hoc* test, *n* = 3 each). Importantly, whole-cell SERT protein levels were not changed significantly by the siRNAs targeting NSF (*F*_(2,14)_ = 1.057; *P* = 0.374, one-way ANOVA, *n* = 5 to 6 each) (Figure 3C,D). To investigate the effect of NSF on SERT uptake function, we conducted a fluorescence-based uptake assay in HEK293-hSERT cells. As shown in Figure 4, both NSF siRNAs decreased fluorescence uptake (siRNA-1; *P* = 0.005 and siRNA-2; *P* < 0.001, one-way repeated measures ANOVA with Tukey’s *post hoc* test, *n* = 8 each). Fluoxetine completely inhibited uptake (Figure 4), including nonspecific uptake.

**Figure 5:**
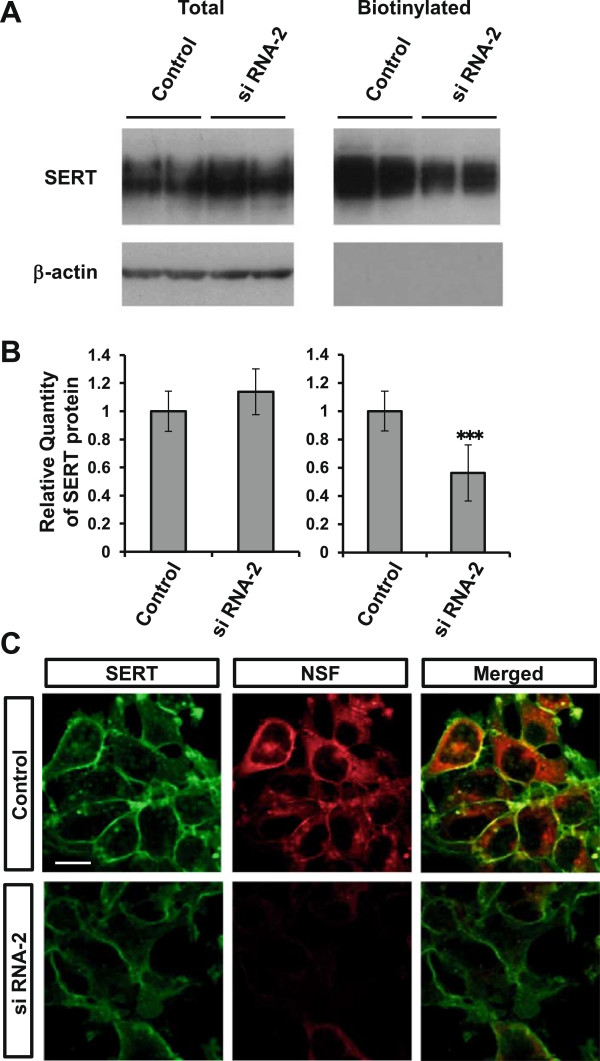
**NSF knockdown results in decreased SERT expression at the plasma membrane in HEK293-hSERT cells. (A)** Biotinylation experiments in HEK293-hSERT cells transfected with siRNA-2 targeting a specific NSF sequence or negative control. Transfected cells were incubated with sulfo-NHS-SS-biotin, and labeled proteins were analyzed by immunoblotting using anti-SERT antibodies. **(B)** Quantitation of relative band densities for SERT was performed by scanning densitometry. Data are expressed as the means ± standard deviation, *n* = 6 to 9. ***P < 0.001 vs negative control (two-tailed unpaired *t*-test). **(C)** Double immunocytochemical staining for SERT (green) and NSF (red) in HEK293-hSERT cells transfected with control siRNA (upper panels) and siRNA for NSF (siRNA-2, lower panels). Scale bar: 10 μm. Results are representative of three independent experiments. NSF, *N*-ethylmaleimide-sensitive factor; SERT, serotonin transporter; siRNA, small interfering RNA.

Next, we conducted biotinylation experiments in HEK293-hSERT cells using sulfo-NHS-SS-biotin. This compound, which binds to lysine and arginine residues in proteins, is cell impermeant and labels cell-surface proteins. Cells transfected with the siRNA of NSF (siRNA-2) or a negative control were incubated with sulfo-NHS-SS-biotin, followed by isolation of labeled proteins with avidin beads and analysis by Western blotting using anti-SERT antibodies. For the biotinylated membrane fraction, after Western blot analysis, the membrane was stained with CBB as a protein-loading control (Additional file 4: Figure S4). As shown in Figure 5A,B, the level of SERT protein at the cell membrane was decreased by an average of 50% (*t* = 5.399; *df* = 16; *P* < 0.001, two-tailed unpaired *t*-test, *n* = 9) following NSF knockdown, despite no change in the total levels of SERT protein (*t* = -1.565; *df* = 10; *P* = 0.149, two-tailed unpaired *t*-test, *n* = 6). Finally, we examined the distribution of SERT in HEK293-hSERT cells when NSF was suppressed. In support of the results of the experiment using sulfo-NHS-SS-biotin, the membrane expression of SERT was decreased by NSF knockdown in HEK293-hSERT cells (Figure 5C).

### Association between serotonin transporter and *N*-ethylmaleimide-sensitive factor *in vivo*

To determine the physiological significance of our findings *in vivo*, we examined: (a) the interaction between SERT and NSF in the mouse brain by immunoprecipitation and Western blotting and (b) the cellular distributions of NSF and SERT in cultured mouse raphe neurons by immunocytochemistry and microscopy.

**Figure 6:**
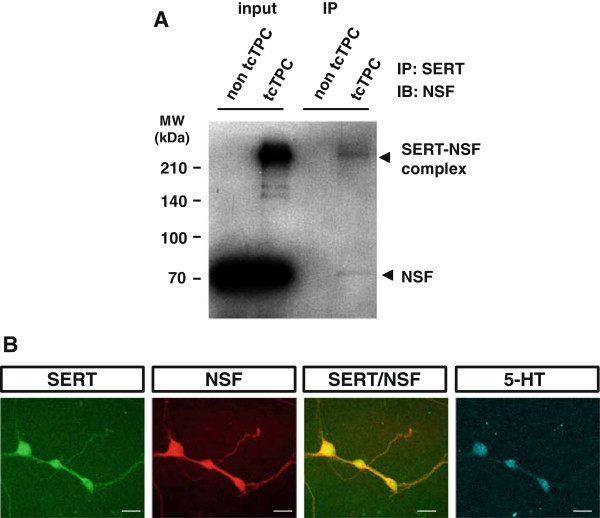
**NSF interacts with SERT *in vivo*. (A)** Interaction of SERT with NSF in mouse brain. Immunoblot of total proteins from non-tcTPC- and tcTPC-treated mouse brains (as input, lanes 1 and 2, respectively). Proteins from non-tcTPC- or tcTPC-treated mouse brains were immunoprecipitated with SERT antibodies (lane 3 and 4), and the resulting immunoblot was probed for NSF. In immunoprecipitated samples using tcTPC-treated mouse brains, SERT–NSF complexes and free NSF were identified (lane 4). Results are representative of three independent experiments. **(B)** NSF co-localizes with SERT in primary cultures of mouse raphe nuclei neurons. Triple immunocytochemical staining for SERT (green), NSF (red) and 5-HT (blue) in primary cultures of mouse raphe nuclei neurons. The third panel (merged) shows that NSF co-localizes with SERT primary cultures of mouse raphe nuclei neurons. These neurons are 5-HT-positive serotonergic neurons (as shown in the fourth panel). Scale bars: 10 μm. Results are representative of three independent experiments. 5-HT, 5-hydroxytryptamine; IB, immunoblotting; IP, immunoprecipitation; MW, molecular weight; NSF, *N*-ethylmaleimide-sensitive factor; SERT, serotonin transporter; tcTPC, time-controlled transcardiac perfusion cross-linking.

Schmitt-Ulms and colleagues have established a method that covalently conserves protein interactions through tcTPC [40]. This method enables the preservation of protein–protein interactions that occur under physiological conditions. We investigated the interaction of SERT with NSF in the mouse brain using this tcTPC method. First, we examined the accuracy of the method. Total protein from non-tcTPC- or tcTPC-treated mouse brains was analyzed by immunoblotting, and we confirmed that SERT-containing cross-linked complexes were retained by this method (see Additional file 5: Figure S5A). Second, we checked whether the complexes were precipitated by anti-SERT antibodies and confirmed that SERT-containing cross-linked complexes were precipitated in a dose-dependent manner using this antibody (see Additional file 5: Figure S5B). Then, finally, we investigated the binding of SERT to NSF. As shown in Figure 6A, NSF co-immunoprecipitated with SERT from tcTPC-treated brain cells indicating that NSF interacts with SERT in the mouse brain under physiological conditions. Next, the cellular distributions of NSF and SERT in cultured mouse raphe neurons were examined. About 10% of all cultured cells were 5-HT-positive neurons in support of a previous report (data not shown) [36]. NSF was ubiquitously expressed in all cultured cells (data not shown). As shown in Figure 6B, triple immunocytochemical staining for SERT, NSF and 5-HT revealed that NSF co-localizes with SERT in the cell body and fibers of cultured serotonergic neurons.

### SLC6A4 and *N*-ethylmaleimide-sensitive factor expression in the raphe region of post-mortem brains from autism patients

**Figure 7:**
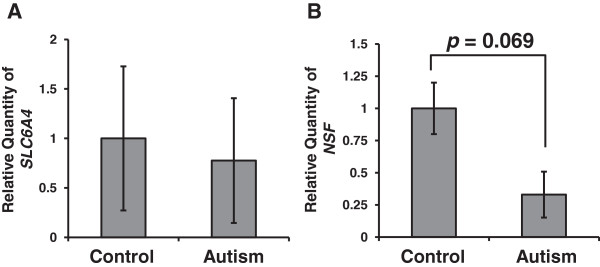
***SLC6A4 *and *NSF *expression in the raphe region of post-mortem brains.** Comparison of *SLC6A4***(A)** and *NSF***(B)** expression levels in the raphe region of post-mortem brains from control and autistic subjects. The Mann–Whitney *U* test was used to compare gene expression levels between autism and control groups. Data are presented as the means ± standard error of the mean. *n* = 11 control and *n* = 7 autism. NSF, *N*-ethylmaleimide-sensitive factor.

The demographic characteristics of subjects (seven with autism and eleven control subjects) are described in Tables 2 and 3. There were no significant differences in age (*P* = 1.000, Mann–Whitney *U* test), race (*P* = 0.305, Fisher’s exact test), gender (*P* = 0.596, Fisher’s exact test) and PMI (*P* = 0.513, Mann–Whitney *U* test) between the autism and control groups (Table 3). Although changes in SERT function and expression have been implicated in autism, mRNA expression of the *SLC6A4* gene that encodes SERT in the brains of autistic individuals has never been reported. Therefore, first, we measured *SLC6A4* expression in the raphe region of post-mortem brains from autistic individuals and controls using qRT-PCR. *SLC6A4* expression was normalized to the expression levels of an internal control (*ACTB*). As shown in Figure 7A, there are wide individual differences in the expression level of *SLC6A4* among the subjects, and the level did not differ significantly between subjects with autism and controls (*P* = 0.928, Mann–Whitney *U* test). Then, we measured *NSF* expression in the same way. *NSF* expression was normalized to the expression of *ACTB*. We found that the *NSF* expression level in autism patients tended to be lower than that in controls; however, this trend was not statistically significant (*P* = 0.069, Mann–Whitney *U* test) (Figure 7B).

**Table 2:** Information for post-mortem brain tissues

**Sample ID**	**Diagnosis**	**Age (years)**	**Gender**	**Post-mortem interval (hours)**	**Race**	**Cause of death**
1065	Control	15	M	12	Caucasian	Multiple injuries
1297	Control	15	M	16	African-American	Multiple injuries
1407	Control	9	F	20	African-American	Asthma
1541	Control	20	F	19	Caucasian	Head injuries
1708	Control	8	F	20	African-American	Asphyxia, multiple injuries
1790	Control	14	M	18	Caucasian	Multiple injuries
1793	Control	12	M	19	African-American	Drowning
1860	Control	8	M	5	Caucasian	Cardiac arrhythmia
4543	Control	29	M	13	Caucasian	Multiple injuries
4638	Control	15	F	5	Caucasian	Chest injuries
4722	Control	14	M	16	Caucasian	Multiple injuries
797	Autism	9	M	13	Caucasian	Drowning
1638	Autism	20	F	50	Caucasian	Seizure
4231	Autism	8	M	12	African-American	Drowning
4721	Autism	8	M	16	African-American	Drowning
4899	Autism	14	M	9	Caucasian	Drowning
5000	Autism	27	M	8.3	NA	NA
6294	Autism	16	M	NA	NA	NA

F, female; M, male; NA, not available.

**Table 3:** Demographic data associated with raphe brain-tissue samples

	**Control (*n*** **= 11)**	**Autism (*n*** **= 7)**	***P *** **value**
Age (years) (range)	14.45 (8–29)	14.57 (8–27)	NS^a^
Race, *n* (%)	Caucasian 7 (63.6), African-American 4 (36.4)	Caucasian 3 (42.9), African-American 2 (28.6), NA 2 (28.6)	NS^b^
Gender, *n* (%)	Male 7 (63.6), Female 4 (36.4)	Male 6 (85.7), Female 1 (14.3)	NS^b^
Post-mortem interval (hours) (range)	14.82 (5–20)	18.05 (8.3-50)	NS^a^

^a^Derived from Mann–Whitney *U* test, ^b^Derived from Fisher’s exact test.

NA, not available; NS, not significant.

### SLC6A4 and *N*-ethylmaleimide-sensitive factor expression in lymphocytes from patients with autism spectrum disorders

**Figure 8:**
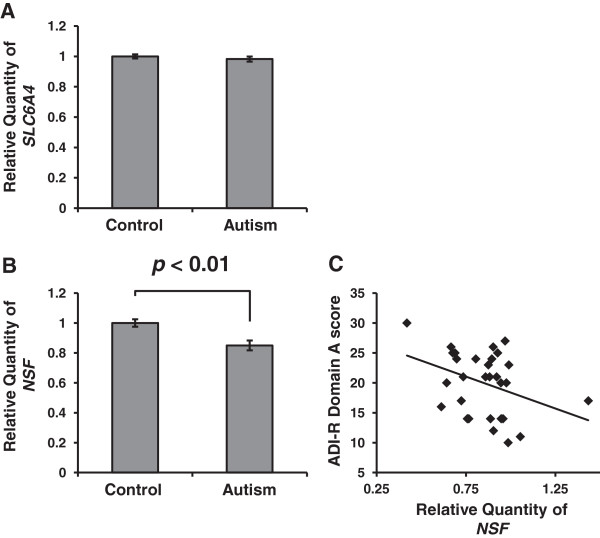
***SLC6A4 *and *NSF *expression in lymphocytes.** Comparison of *SLC6A4***(A)** and *NSF***(B)** expression levels in lymphocytes from control and ASD subjects. The Mann–Whitney *U* test was used to compare gene expression levels between autism and control groups. Data are presented as the means ± standard error of the mean. *n* = 30 control and *n* = 30 autism. The *NSF* expression levels in ASD patients were significantly lower than in controls (*P* = 0.0011). **(C)** Correlation between lymphocyte *NSF* expression levels and Autism Diagnostic Interview-Revised (ADI-R) domain A scores in autistic subjects. There was a negative correlation between lymphocyte *NSF* expression levels and ADI-R domain A scores (*r*_*s*_ = 0.131, *P* = 0.0498), *n* = 30 autism. ADI-R, autism diagnostic interview-revised; NSF, *N*-ethylmaleimide-sensitive factor.

NSF is expressed ubiquitously in all normal human tissues including lymphocytes [59]. Lymphocytes also carry SERT [60]. Thus, we measured expressions of these genes in lymphocytes from individuals with ASD and age- and sex-matched controls by qRT-PCR. The demographic characteristics of the subjects (30 with ASD and 30 control subjects) are described in Table 4. There were no significant differences in age (*P* = 0.928, Mann–Whitney *U* test) or IQs (verbal IQ, *P* = 0.098, Mann–Whitney *U* test; performance IQ, *P* = 0.076, Mann–Whitney *U* test; full-scale IQ, *P* = 0.554, Mann–Whitney *U* test) between the ASD and control groups (Table 4). As shown in Figure 8A, the expression level of *SLC6A4* did not differ significantly between subjects with ASD and controls (*P* = 0.518, Mann–Whitney *U* test). On the other hand, we found that the *NSF* expression level in ASD patients were significantly lower than that in controls (*P* = 0.0011, Mann–Whitney *U* test) (Figure 8B). Moreover, there was a significantly negative correlation between *NSF* expression and ADI-R Domain A score, which quantified impairment in social interaction, in individuals with ASD (*r*_*s*_ = 0.131, *P* = 0.0498, Spearman’s rank correlation coefficient test) (Figure 8C). There were no significant correlations between *NSF* expression levels and levels of *SLC6A4* and any other symptom profile or clinical variables (data not shown).

**Table 4:** Demographic data associated with lymphocyte samples

	**Control (*N*** **= 30)**^**b**^	**Autism (*N*** **= 30)**^**b**^	***P *** **value**
Age (years)	11.1 ± 2.3 (6–16)	11.6 ± 2.7 (7–16)	NS^a^
ADI-R			
Domain A score		20.0 ± 5.3 (10–30)	
Domain BV score		14.3 ± 4.0 (8–23)	
Domain C score		8.5 ± 3.4 (3–9)	
Domain D score		3.1 ± 1.1 (1–5)	
WISC-III			
Verbal IQ	99.1 ± 10.3 (77–120)	90.4 ± 28.7 (44–153)	NS^a^
Performance IQ	97.0 ± 10.2 (76–114)	89.8 ± 22.9 (47–131)	NS^a^
Full-scale IQ	97.8 ± 9.5 (82–115)	89.0 ± 26.9 (42–140)	NS^a^

^a^Derived from Mann–Whitney *U* test; ^b^values are expressed as mean ± standard deviation (range).

ADI-R, Autism Diagnostic Interview-Revised; IQ, intelligence quotient; NS, not significant; WISC-III, the third edition of the Wechsler Intelligence Scale for Children.

## Discussion

In this study, NSF was identified as a novel SERT-binding protein interacting with the N-terminal region of SERT. NSF knockdown resulted in decreased membrane expression of SERT and decreased uptake of substrate. These results clearly show that NSF modulates SERT membrane trafficking, which is consistent with its uptake function. An immunoprecipitation assay using mouse brain and immunocytochemistry of cultured mouse raphe neurons clearly indicated that SERT–NSF complexes were formed under physiological conditions *in vivo*. In addition, a study of post-mortem brains revealed that the *SLC6A4* expression level was not affected in subjects with autism, but the *NSF* expression level in the raphe region tended to be decreased; however, this potential trend is not statistically significant. In lymphocytes, the *SLC6A4* expression level was also unchanged, but the *NSF* expression level was significantly decreased in subjects with ASD and correlated with the severity of clinical symptoms.

### *N*-ethylmaleimide-sensitive factor functions and protein binding

NSF is a homohexameric ATPase [61,62], which is an essential component of the protein machinery responsible for various membrane fusion events, including intercisternal Golgi protein transport and the exocytosis of synaptic vesicles [63]. NSF binds to soluble NSF attachment protein–receptor (SNARE) complexes and mediates the recycling of spent SNARE complexes for subsequent rounds of membrane fusion [63,64]. While this is a major function of NSF, it also interacts with receptor proteins, such as AMPA, β2 adrenergic and GABA_A_ receptors, and is thought to affect their trafficking patterns or recycling [49-57]. Additionally, an interaction between NSF and arrestin 1 regulates the expression of vesicular glutamate transporter 1 and excitatory amino acid transporter 5 in the photoreceptor synapse [58]. In the present study, we found, for the first time, that NSF binds the neurotransmitter transporter SERT and regulates its function in the CNS.

### Serotonin transporter forms complexes with *N*-ethylmaleimide-sensitive factor *in vivo*

Several putative SERT-binding proteins have been reported [21-32]. However, almost all of these were identified using the yeast two-hybrid system and little is known regarding whether any of these proteins bind to SERT and regulate its function in the mammalian brain. Also, little is known about the involvement of these proteins in autism [65,66]. Therefore, in this study, we used a pull-down system together with mouse brain tissue to identify novel SERT-binding proteins. Moreover, we used the tcTPC method, which is an innovative tool for studying proteins in living tissues [40]. This method enabled us to preserve protein–protein interactions occurring under physiological conditions. This cross-linking also preserves membrane protein assemblies, which are degraded by solubilizing detergents. For instance, whereas most detergents cause rapid disintegration of the γ-secretase complex, three of four known components of the complex were purified and identified from harsh detergents and a high salt concentration by tcTPC [40]. Because NSF was not co-immunoprecipitated with SERT from non-tcTPC-treated brains (Figure 6A), it is likely that SERT–NSF complexes are sensitive to solubilizing detergents. The discovery of complexes including NSF and SERT, which form in the mammalian brain under physiological conditions, in the present study, is important from the viewpoint of their potential involvement in the pathophysiology of disorders such as autism. It is not yet clear whether NSF binds SERT directly or indirectly. In addition, the band for the SERT–NSF complex was smeared, suggesting that multiple types of SERT–NSF complexes exist. It is possible that SERT interacts with NSF through other proteins. Indeed, it is possible that GABA_A_ receptors interact with NSF via GABA_A_ receptor-associated protein, and regulate its intracellular distribution and recycling [56,67]. Detailed analyses of these SERT–NSF complexes are needed.

### Serotonin transporter and *N*-ethylmaleimide-sensitive factor expressions in autism

Recently, Nakamura and colleagues reported that the levels of SERT based on its radioligand binding were significantly lower throughout the brain in autistic individuals compared with controls [17]. On the other hand, Azmitia and colleagues reported increased immunoreactivity to a SERT antibody of serotonin axons in the post-mortem cortex of autism patients [18]. Our results show that, at least, *SLC6A4* mRNA expression is normal in the raphe region of post-mortem brains from subjects with autism. Our findings and previous results lead us to two suggestions. First, although the transcription of *SLC6A4* is normal in subjects with autism, the level of SERT protein at the pre-synaptic membrane is decreased because of an impairment of the trafficking system. Second, SERT protein that is not delivered to the pre-synaptic membrane accumulates in axon fibers in the brains of subjects with autism. In lymphocytes, we found that *SLC6A4* expression was not changed in subjects with ASD. In contrast with our finding, Hu *et al*. previously reported that there was a significant decrease in the expression in the more severely affected twin for autistic twin pairs studied using lymphoblastoid cell lines [68]. This study used lymphoblastoid cell lines, not lymphocytes, from only three sets of discordant twins, and *SLC6A4* expression was not compared with normal controls [68]. These differences may be the cause of the discrepancies between the present study and that report.

We found that the *NSF* expression levels tended to decrease in the raphe region of post-mortem brains from subjects with autism; however, this trend was not statistically significant (*n* = 11 control and *n* = 7 autism). Further studies with larger numbers of post-mortem brains are needed to clarify *NSF* expression status in the brain of autism patients. In lymphocytes, we found, for the first time, that *NSF* expression was significantly lower in subjects with ASD and lower *NSF* expression correlated with the severity of impairments in social interaction. Our findings suggest that peripheral NSF mRNA levels may serve as a reliable peripheral biological marker of ASD.

Sullivan *et al*. reported that the expression levels of a number of biologically relevant genes are statistically similar between lymphocytes and CNS tissues including the brain, and suggested that the cautious and thoughtful use of lymphocytic gene expression may be a useful surrogate for gene expression in the CNS when it has been determined that the gene is expressed in both [69]. In support of previous findings [59,60], the expressions of *SLC6A4* and *NSF* were detected in both tissues, and it is likely that levels of *SLC6A4* and *NSF* in the peripheral lymphocytes may reflect the levels in post-mortem brains, although further study is needed.

### The serotonin transporter–*N*-ethylmaleimide-sensitive factor binding and implications for pathophysiology in autism

Sanyal and Krishnan reported a lethal mutation in the *Drosophila* homolog of NSF [70]. Intriguingly, mutant adult survivors show abnormal seizure-like paralytic behavior [70]. Additionally, Matveeva and colleagues reported that decreased production of NSF is associated with epilepsy in rats [71]. Importantly, a high rate of co-occurrence of autism and epilepsy has been described [72-76]. Approximately 30% of children with autism have epilepsy and 30% of children with epilepsy have autism [77]. Interestingly, an abnormal status for SERT has been reported in epileptic patients as follows. Autoradiography experiments have revealed that the temporal neocortex surrounding the epileptic focus of patients with mesial temporal lobe epilepsy presents diminished SERT binding in all cortical layers [78]. A significant decrease was found in the SERT density in the platelet membranes from epileptic patients having undergone an epileptic seizure [79,80]. Additionally, it has been shown that epileptic patients who had been treated with inhibitors of serotonin reuptake, such as fluoxetine and citalopram, in addition to their ongoing antiepileptic therapy displayed remarkable clinical improvements [81,82]. This indirect evidence implies the relationship between SERT and NSF in neurological disorders, such as autism. Further investigations of the status of SERT–NSF binding in the brain of autism patients would be useful for understanding the mechanisms that underlie autism. In addition, an animal model, such as an NSF conditional knockout mouse, would be a useful tool for understanding the mechanisms that underlie ASD.

As mentioned above, NSF interacts with neurotransmitter receptors such as AMPA, β2 adrenergic and GABA_A_ receptors, and regulates the membrane trafficking and recycling of these receptors [49-57]. An abnormal status of many of these receptors has been reported in autism. Binding of GABA_A_α5 and its radioligand was significantly lower throughout the brains of participants with ASDs compared with controls [83]. The mRNA levels of AMPA receptor were significantly increased in the post-mortem cerebellum of autistic individuals, while the receptor density was slightly decreased in people with autism [84]. It is possible that NSF may contribute to the pathophysiology of autism through these known interactions with relevant molecules.

## Conclusions

This study showed that dysfunctional trafficking of SERT mediated by NSF may be linked with the pathophysiology of autism. The identification of SERT-binding proteins provides new opportunities not only to dissect the accessory components involved in SERT function and regulation, but also to elucidate the pathophysiology of psychiatric disorders or developmental disorders, such as autism. Future studies should examine the pathophysiological implications of SERT–NSF interactions for autism.

## Abbreviations

5-HT: 5-hydroxytryptamine; ADI-R: autism diagnostic interview-revised; ANOVA: analysis of variance; ASD: autism spectrum disorder; cDNA: complementary DNA; CNS: central nervous system; C-SERT: C-terminal domain of SERT; DMEM: Dulbecco's modified Eagle's medium; GST: glutathione S-transferase; HBSS: Hank’s balanced salt solution; Hic-5: hydrogen peroxide-inducible clone 5 protein; hSERT: human serotonin transporter; IQ: intelligence quotient; LC-MS/MS: liquid chromatography-tandem mass spectrometry; MacMARCKS: macrophage myristoylated alanine-rich C kinase substrate; MTT: 3-(4,5-dimethylthiazol-2-yl)-2,5-diphenyl tetrazolium bromide; MW: molecular weight; nNOS: nitric oxide synthase; NSF: *N*-ethylmaleimide-sensitive factor; N-SERT: N-terminal domain of SERT; PBS: phosphate-buffered saline; PCR: polymerase chain reaction; PMI: post-mortem interval; PP2A: phosphatase 2A; qRT-PCR: quantitative real-time reverse-transcription-polymerase chain reaction; RT: room temperature; RT-PCR: reverse-transcription-polymerase chain reaction; SCAMP2: secretory carrier membrane protein 2; SCID: structured clinical interview for DSM-IV; SERT: serotonin transporter; siRNA: small interfering RNA; SLC6A4: member 4 of solute carrier family 6 (neurotransmitter transporter); SNARE: soluble NSF attachment protein–receptor; tcTPC: time-controlled transcardiac perfusion cross-linking.

## Competing interests

The authors declare that they have no competing interests.

## Authors’ contributions

HM and TK co-designed the study. KI and HM collected blood samples; collected, analyzed and interpreted the data and prepared the manuscript. TT and SY produced the SERT antibody. KO, HT, KY and SM collected, analyzed and interpreted the data. KN recruited participants, collected blood samples and obtained post-mortem brain samples. KJT and KM collected blood samples and undertook clinical evaluations. MT recruited participants. TS recruited participants and diagnosed ASD. TK collected, analyzed and interpreted the data and prepared the manuscript. NM analyzed and interpreted the data, and prepared the manuscript. All authors read and approved the final manuscript.

## Supplementary Material

Additional file 1: Figure S1 N-tail-specific binding of syntaxin-1A to SERT was confirmed by Western blot analysis. Click here for file

Additional file 2: Figure S2 SERT is transported to the plasma membrane in HEK293-hSERT cells. **(A, B)** Double immunocytochemical staining for SERT (green) and the membrane maker cadherin (red) in HEK293-hSERT cells. **(C)** SERT was mainly co-localized with the membrane maker (cadherin) (merged). Scale bar: 10 μm. Results are representative of three independent experiments. Click here for file

Additional file 3: Figure S3 Transfection efficacy of siRNA in HEK293-hSERT cells. We determined the proportion of siRNA-transfected HEK293-hSERT cells using a commercially available fluor-oligo kit (TYE 563 DS, Integrated DNA Technologies). The proportion of siRNA-transfected cells was 90%. Upper panels show untreated cells and lower panels show red fluorescent oligo-transfected cells. Left panels show phase-contrast images and right panels show the images obtained by fluorescence microscopy (excitation: 546 nm, emission: 590 nm). Scale bar: 50 μm. Results are representative of three independent experiments. Click here for file

Additional file 4: Figure S4 CBB staining of membranes from biotinylated fractions. Biotinylation experiments in HEK293-hSERT cells transfected with siRNA-2 targeting a specific NSF sequence or negative control. Transfected cells were incubated with sulfo-NHS-SS-biotin. After Western blot analysis, the membrane was stained with CBB as a protein-loading control. Click here for file

Additional file 5: Figure S5 Confirmation of tcTPC efficacy. **(A)** Western blotting of total proteins from non-tcTPC- or tcTPC-treated mouse brains (lanes 1 and 2, respectively) using anti-SERT antibodies. Results are representative of three independent experiments. It was confirmed that SERT-containing cross-linked complexes were retained by the tcTPC method (lane 2). **(B)** Proteins from non-tcTPC- or tcTPC-treated mouse brains were immunoprecipitated with rat immunoglobulin G (IgG) as a negative control (lanes 1 and 5) and SERT antibodies (lanes 2 to 4 and 6 to 8), and the resulting Western blot was probed for SERT. In immunoprecipitated samples using tcTPC-treated mouse brains, SERT-containing cross-linked complexes were identified (lanes 6 to 8) in a dose-dependent manner. Results are representative of three independent experiments. Click here for file
