# Supplementary material for: N-ethylmaleimide-sensitive factor interacts with the serotonin transporter and modulates its trafficking: implications for pathophysiology in autism
Source: Mol Autism. 2014 May 10;5:33. doi: 10.1186/2040-2392-5-33 (PMC4022412; doi:10.1186/2040-2392-5-33)
Supplement: Additional file 1: Figure S1 — N-tail-specific binding of syntaxin-1A to SERT was confirmed by Western blot analysis. [file 2040-2392-5-33-S1.pdf]

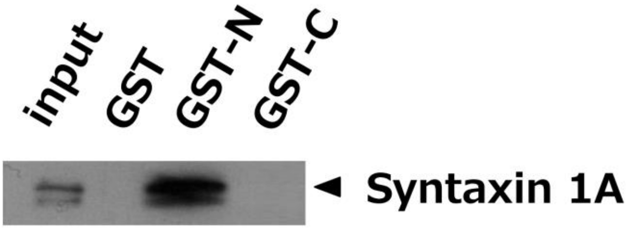

Additional file 1. N-tail-specific binding of Syntaxin 1A to SERT was confirmed by western blot analysis.
