# Supplementary material for: N-ethylmaleimide-sensitive factor interacts with the serotonin transporter and modulates its trafficking: implications for pathophysiology in autism
Source: Mol Autism. 2014 May 10;5:33. doi: 10.1186/2040-2392-5-33 (PMC4022412; doi:10.1186/2040-2392-5-33)
Supplement: Additional file 2: Figure S2 — SERT is transported to the plasma membrane in HEK293-hSERT cells. (A, B) Double immunocytochemical staining for SERT (green) and the membrane maker cadherin (red) in HEK293-hSERT cells. (C) SERT was mainly co-localized with the membrane maker (cadherin) (merged). Scale bar: 10 μm. Results are representative of three independent experiments. [file 2040-2392-5-33-S2.pdf]

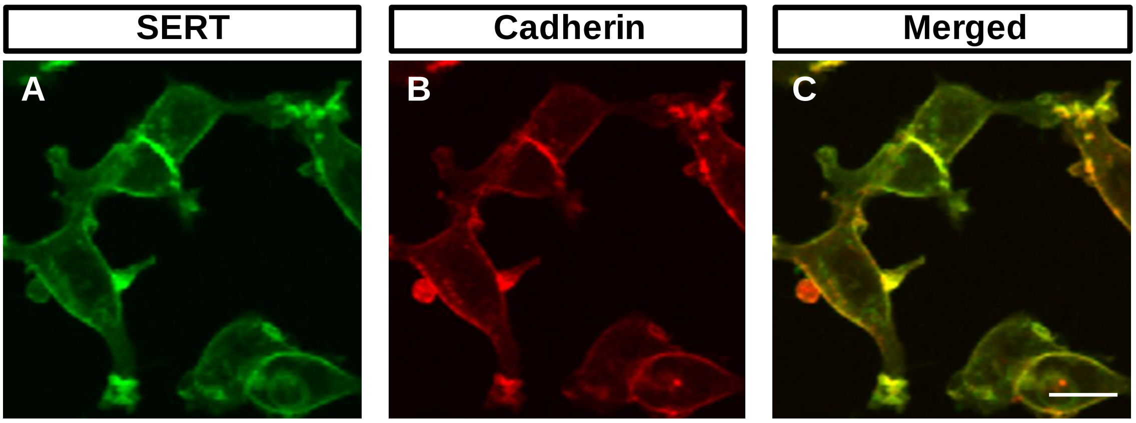

Additional file 2. SERT is transported to the plasma membrane in HEK293-hSERT cells. (A-C) Double immunocytochemical staining for SERT (green) and the membrane maker cadherin (red) in HEK293-hSERT cells. SERT mainly colocalized with the membrane maker (cadherin) (C: merged). Scale bar, 10 μm. Results are representative of three independent experiments.
