# Supplementary material for: N-ethylmaleimide-sensitive factor interacts with the serotonin transporter and modulates its trafficking: implications for pathophysiology in autism
Source: Mol Autism. 2014 May 10;5:33. doi: 10.1186/2040-2392-5-33 (PMC4022412; doi:10.1186/2040-2392-5-33)
Supplement: Additional file 3: Figure S3 — Transfection efficacy of siRNA in HEK293-hSERT cells. We determined the proportion of siRNA-transfected HEK293-hSERT cells using a commercially available fluor-oligo kit (TYE 563 DS, Integrated DNA Technologies). The proportion of siRNA-transfected cells was 90%. Upper panels show untreated cells and lower panels show red fluorescent oligo-transfected cells. Left panels show phase-contrast images and right panels show the images obtained by fluorescence microscopy (excitation: 546 nm, emission: 590 nm). Scale bar: 50 μm. Results are representative of three independent experiments. [file 2040-2392-5-33-S3.pdf]

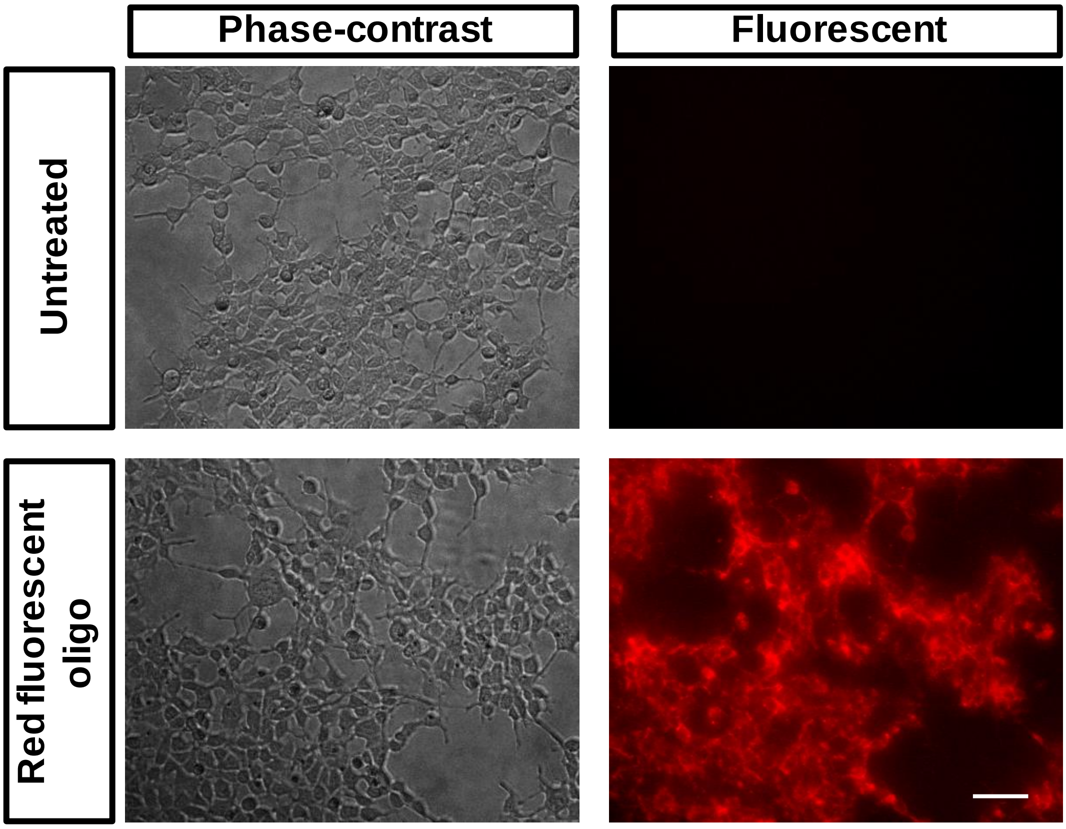

Additional file 3. Transfection efficacy of siRNA in HEK293-hSERT cells. We determined the proportion of siRNA-transfected HEK293-hSERT cells using a commercially available fluor-oligo (TYE 563 DS, Integrated DNA Technologies). The proportion of siRNA-transfected cells was 90%. Upper panels show untreated cells and lower panels show red fluorescent oligo-transfected cells. Left panels show phase-contrast images and right panels show the images obtained by fluorescence microscopy (excitation: 546 nm, emission: 590 nm). Scale bar, 50 μm. Results are representative of three independent experiments.
