# Supplementary material for: N-ethylmaleimide-sensitive factor interacts with the serotonin transporter and modulates its trafficking: implications for pathophysiology in autism
Source: Mol Autism. 2014 May 10;5:33. doi: 10.1186/2040-2392-5-33 (PMC4022412; doi:10.1186/2040-2392-5-33)
Supplement: Additional file 4: Figure S4 — CBB staining of membranes from biotinylated fractions. Biotinylation experiments in HEK293-hSERT cells transfected with siRNA-2 targeting a specific NSF sequence or negative control. Transfected cells were incubated with sulfo-NHS-SS-biotin. After Western blot analysis, the membrane was stained with CBB as a protein-loading control. [file 2040-2392-5-33-S4.pdf]

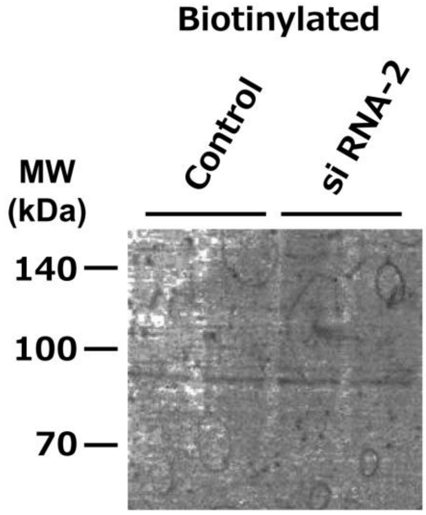

Additional file 4. CBB staining of membrane from biotinylated fractions. Biotinylation experiments in HEK293-hSERT cells transfected with siRNA-2 targeting a specific NSF sequence or negative control. Transfected cells were incubated with sulfo-NHS-SS-biotin. After western blot analysis, the membrane was stained with CBB as protein loading control.
