# Supplementary material for: N-ethylmaleimide-sensitive factor interacts with the serotonin transporter and modulates its trafficking: implications for pathophysiology in autism
Source: Mol Autism. 2014 May 10;5:33. doi: 10.1186/2040-2392-5-33 (PMC4022412; doi:10.1186/2040-2392-5-33)
Supplement: Additional file 5: Figure S5 — Confirmation of tcTPC efficacy. (A) Western blotting of total proteins from non-tcTPC- or tcTPC-treated mouse brains (lanes 1 and 2, respectively) using anti-SERT antibodies. Results are representative of three independent experiments. It was confirmed that SERT-containing cross-linked complexes were retained by the tcTPC method (lane 2). (B) Proteins from non-tcTPC- or tcTPC-treated mouse brains were immunoprecipitated with rat immunoglobulin G (IgG) as a negative control (lanes 1 and 5) and SERT antibodies (lanes 2 to 4 and 6 to 8), and the resulting Western blot was probed for SERT. In immunoprecipitated samples using tcTPC-treated mouse brains, SERT-containing cross-linked complexes were identified (lanes 6 to 8) in a dose-dependent manner. Results are representative of three independent experiments. [file 2040-2392-5-33-S5.pdf]

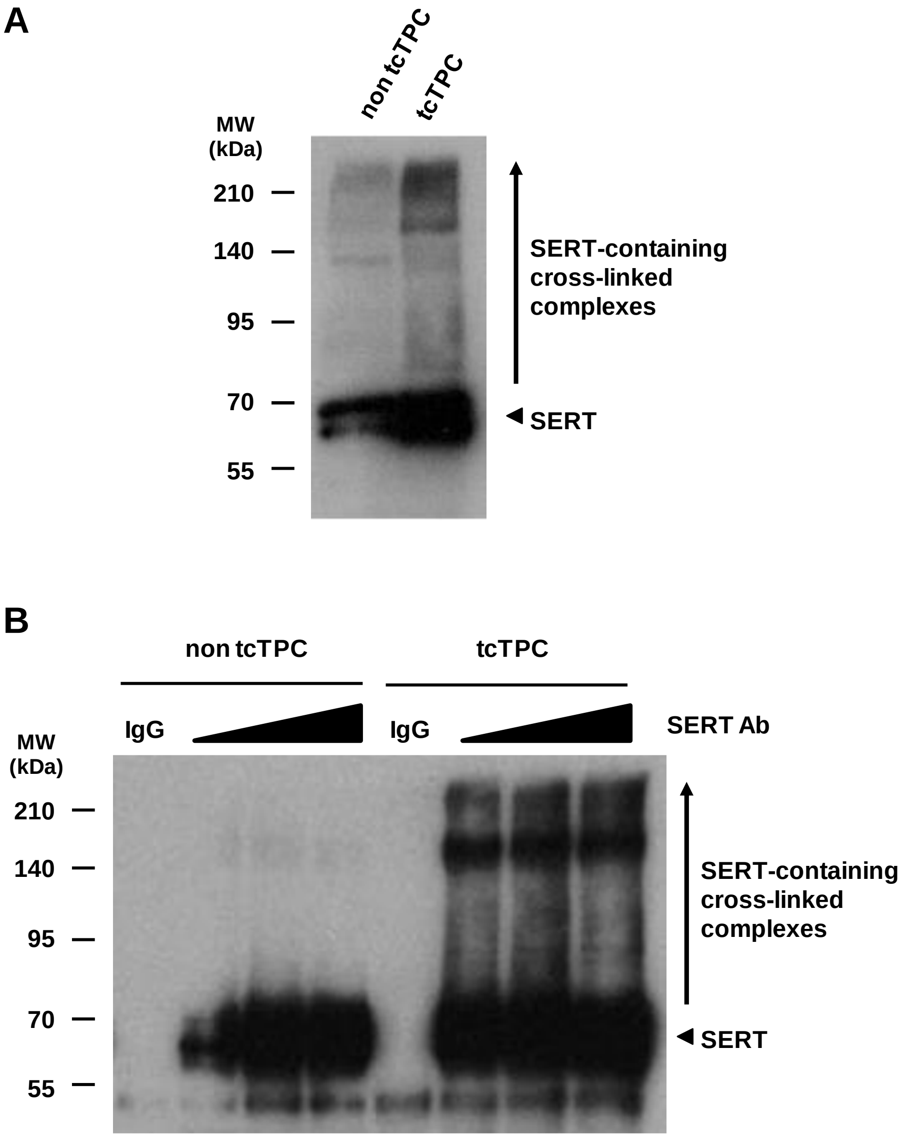

Additional file 5. Confirmation of tcTPC efficacy. (A) Western blotting of total proteins from non tcTPC- or tcTPC-treated mouse brains (*lane 1, 2, respectively*) using anti-SERT antibody. Results are representative of three independent experiments. It was confirmed that SERT-containing cross-linked complexes are retained by the tcTPC method (*lane 2*). (B) Proteins from non tcTPC- or tcTPC-treated mouse brains were immunoprecipitated with rat IgG as a negative control (*lanes 1 and 5, respectively*) and SERT antibody (*lanes 2–4 and 6–8, respectively*), and the resulting Western blot was

probed for SERT. In immunoprecipitated samples using tcTPC-treated mouse brains, SERT-containing cross-linked complexes were identified (*lane 6–8*) in a dose-dependent manner. Results are representative of three independent experiments.
